# Efficacy and safety of mesh reinforced cruroplasty with Phasix™ ST vs. Bio-A^®^: systematic review and bayesian meta-analysis

**DOI:** 10.1007/s10029-026-03666-y

**Published:** 2026-04-15

**Authors:** Michele Manara, Davide Bona, Sara De Bernardi, Marta Cavalli, Quan Wang, Gianluca Bonitta, Davide Guido, Antonio Biondi, Giampiero Campanelli, Luigi Bonavina, Alberto Aiolfi

**Affiliations:** 1 I.R.C.C.S. Ospedale Galeazzi – Sant’Ambrogio, Division of General Surgery, Department of Biomedical Science for Health, University of Milan, Milan, 20122 Italy; 2 University of Insubria, Galeazzi-Sant’Ambrogio Hospital, Milan, Italy; 3 Department of Pharmacy, Health and Nutrition Sciences, University of Calabria (UNICAL), Arcavacata di Rende (Cosenza), Azienda Ospedaliera di Cosenza, Division of General and Foregut Surgery, Cosenza, Italy; 4 National Institute of Gastroenterology – I.R.C.C.S. “Saverio de Bellis”, Division of Data Science, Castellana Grotte, Bari 70013 Italy; 5 G. Rodolico Hospital, Surgical Division, Department of General Surgery and Medical Surgical Specialties, University of Catania, Catania, 95131 Italy

**Keywords:** Hiatal hernia, Bio-A, Phasix ST, Bayesian meta-analysis, Mesh repair, Recurrence

## Abstract

**Purpose:**

Recurrence remains a major challenge after minimally invasive hiatus hernia (HH) repair, particularly in patients with large diaphragmatic defects. Currently, biosynthetic absorbable meshes such as Bio-A^®^ and Phasix™ ST are widely used to reinforce cruroplasty, however evidence comparing their efficacy and safety profile remains limited. This study aims to compare the safety and efficacy of Bio-A^®^ versus Phasix™ ST after minimally invasive HH repair through a Bayesian meta-analysis.

**Methods:**

Systematic search across online databases up to November 2025 was performed. Inclusion criteria targeted elective minimally invasive HH repairs with Bio-A^®^ or Phasix™ ST mesh. Primary outcome was radiologically or endoscopically confirmed anatomical HH recurrence. Overall, severe (Clavien-Dindo ≥ 3), mesh-related complications and reoperation for recurrence were secondary outcomes. A Bayesian hierarchical power prior model was utilised to pool data from both comparative and single-arm studies.

**Results:**

Twenty-one observational studies (2208 patients) were included. Bio-A^®^ was used in 53.1% of cases. The overall recurrence rate was 8.2% (*n* = 170 patients), with a clinical trend toward higher recurrence for Bio-A^®^ vs. Phasix™ ST mesh (10% vs. 6%). The Bayesian meta-analysis showed no statistically significant difference for Bio-A^®^ vs. Phasix™ ST (RR = 1.29, 95% HPD 0.01–8.15). Notably, 38 over 170 patients (17%) required reoperation for HH recurrence with a trend toward higher rates for Bio-A^®^ vs. PhasixTM (36.3% vs. 19.3%). Overall (RR = 1.16, 95% HPD 0.01–8.75) and severe postoperative complications (RR = 1.06, 95% HPD 0.01–12.33) were comparable. Mesh-related complication with esophageal fibrosis occurred in one Bio-A^®^ patient (0.04%).

**Conclusion:**

Bio-A^®^ and Phasix™ ST synthetic absorbable meshes seem safe and effective for hiatal reinforcement with apparently similar rates of recurrence and postoperative morbidity. The choice between these materials should be guided by surgeon preferences and cost-effectiveness.

**Supplementary Information:**

The online version contains supplementary material available at 10.1007/s10029-026-03666-y.

## Introduction

Hiatus hernia (HH) represents a chronic, multifactorial condition characterized by disruption of the physiological antireflux barrier and lower esophageal sphincter competence. Its prevalence among the adult population is estimated between 15 and 20%, with types III and IV accounting for approximately 5–10% of cases of the general population [1, 2]. Minimally invasive repair is currently regarded as the standard treatment modality for symptomatic HH. Elective surgery is also advocated in asymptomatic individuals with large hernia defects due to potential risk of severe complications [3]. Surgical management typically involves extensive esophageal dissection, excision of the hernia sac, tension-free cruroplasty, and fundoplication. Despite surgical intervention, radiological recurrence rates after primary repair remain high, reported to reach up to 66% [4]. To reduce both anatomical and clinical recurrence, reinforcement of the esophageal hiatus with various techniques and mesh materials has been reported. While the use of non-absorbable mesh demonstrated encouraging results, evidence has raised concerns regarding the potential for mesh-related severe complications such as visceral fibrosis and full thickness erosion [5, 6].

By contrast, absorbable synthetic meshes have been linked to minimized complication and decreased short- to mid-term recurrence compared to simple suture repair [7]. Nevertheless, the definitive role of absorbable meshes remains contentious due to limited long-term outcome data. At present, two fully resorbable synthetic meshes are predominantly employed: Bio-A^®^ (Gore Medical, Newark, DE, USA) and Phasix™ ST (C.R. Bard, Inc./Davol, Inc., Warwick, RI, USA) [8]. Bio-A^®^ consists of a three-dimensional polymeric matrix composed of polyglycolic acid and trimethylene carbonate, which undergoes gradual absorption over six months, subsequently replaced by vascularized soft tissue. In contrast, Phasix™ ST is fabricated from poly-4-hydroxybutyrate (P4HB), a polymer degraded in vivo via hydrolytic and enzymatic mechanisms, featuring a unilateral hydrogel barrier (Sepra Technology) designed to reduce adhesion formation to abdominal viscera. Complete resorption and tissue incorporation occur within 12 to 18 months.

While prior single-arm studies have been published, comparative data evaluating outcomes between Bio-A^®^ and Phasix™ ST for crural reinforcement in HH repair remain scarce. This systematic review and meta-analysis aim to critically assess and compare the safety and efficacy of minimally invasive HH repair with cruroplasty reinforced using Bio-A^®^ versus Phasix™ ST mesh.

## Materials and methods

A systematic literature review was conducted in accordance with PRISMA guidelines and registered on PROSPERO (CRD420251142216) [9]. A comprehensive search of PubMed, MEDLINE, Scopus, Web of Science, Cochrane Central Library, Google Scholar, and ClinicalTrials.gov was performed in November 2025 [10]. The search strategy employed combinations of the following MeSH terms and keywords: (“hiatal hernia“[tiab] OR reflux[tiab]) AND (hiatoplasty[tiab] OR cruroplasty[tiab]) AND (reinforcement[tiab] OR mesh[tiab] OR “Bio-A^®^“[tiab] OR “Phasix™ ST“[tiab]) AND (recurrence[tiab] OR complications[tiab] OR “mesh-related complications“[tiab]). Articles published up to November 30, 2025, were considered. To minimize the risk of missing relevant studies, references list of included articles and related reviews were manually screened. Ethical approval was not required for this analysis.

### Eligibility criteria

Studies were eligible if they met the following criteria: (1) elective primary or recurrent minimally invasive HH repair with cruroplasty and mesh augmentation with Bio-A^®^ or Phasix™ ST mesh; (2) adult patients (≥ 18 years old); (3) reporting of short- and medium-term outcomes; and (4) published in English. Exclusion criteria were: (1) use of alternative augmentation techniques or meshes; (2) concomitant bariatric procedures; (3) failure to report primary outcome; (4) case reports/series with < 5 patients; and (5) editorials or reviews.

### Selection process

Two independent reviewers (MM, SD) screened studies according to the predefined inclusion and exclusion criteria. After removing duplicates, title and abstracts were screened, followed by full-text review of articles retained from the previous screening phases. A manual search through reference lists from the relevant articles was performed to identify additional pertinent studies. In cases of overlapping populations, published by the same institution or study group, the study with the longer follow-up or the largest sample size was included. Discrepancies were resolved by two additional blind reviewers (AA, DB).

### Data collection process

Three researchers (MM, SD, AA) extracted data covering the following variables: author, publication year, country, study design, mesh type, total number of patients, gender, age, body mass index, HH type, recurrent hernia at baseline, cruroplasty technique, mesh shape, mesh fixation method, combined antireflux procedure, surgical approach, conversion rate, operative time, postoperative complications, mesh-related complications, length of hospital stay, recurrence, redo surgery, and follow-up duration. Data consistency was verified at the end of the process by two authors (GB, DB).

### Outcome of interest and definition

The primary outcome was recurrence, defined as anatomical recurrence confirmed by imaging (swallow study or computed tomography) or endoscopy. Secondary outcomes included overall, severe, mesh-related complications, and reoperation for recurrence. Postoperative complications were classified according to the Clavien-Dindo system [11].

### Quality assessment

Methodological quality was assessed by three independent reviewers (MM, SD, GB) using the ROBINS-I V2 tool and GRADE approach. The following domains were evaluated: confounding bias, selection bias, classification bias, intervention bias, missing data bias, outcomes measurement bias, and reporting bias. Each domain was rated as “Low”, “Moderate”, “Serious”, or “Critical” risk of bias, and an overall judgment was assigned accordingly [12].

### Statistical analysis

A fully Bayesian meta-analysis was performed to estimate the pooled Risk Ratio (RR). We included single-arm studies into double-arm comparative trials by fitting the hierarchical power prior (HPP), using deviance information criterion (DIC) for model selection [13]. We ran 400,000 Monte Carlo Markov Chain (MCMC) iterations, discharging the first 100,000 MCMC as burn-in. This was sufficient to achieve MCMC convergence that was assessed both using graphical inspection of running means, trace plot and all methods included in the R Boa package [14]. The medians of marginal posterior distributions and 95% highest posterior density (HPD) were computed as inferential summary statistics. We judged the estimated parameters statistical significance by whether its 95% HPD involved the null hypothesis value (1.0). The heterogeneity was assessed by directly interpreting between-study standard deviation (τ): values from 0.1 to 0.5 represent “reasonable heterogeneity,” values from 0.5 to 1.0 represent “fairly high heterogeneity” and values over 1.0 represent “fairly extreme heterogeneity” [15]. All analyses were performed by JAGS software, using R2jags package and R-Cran [16].

## Results

### Literature search & quality assessment

**Fig. 1:**
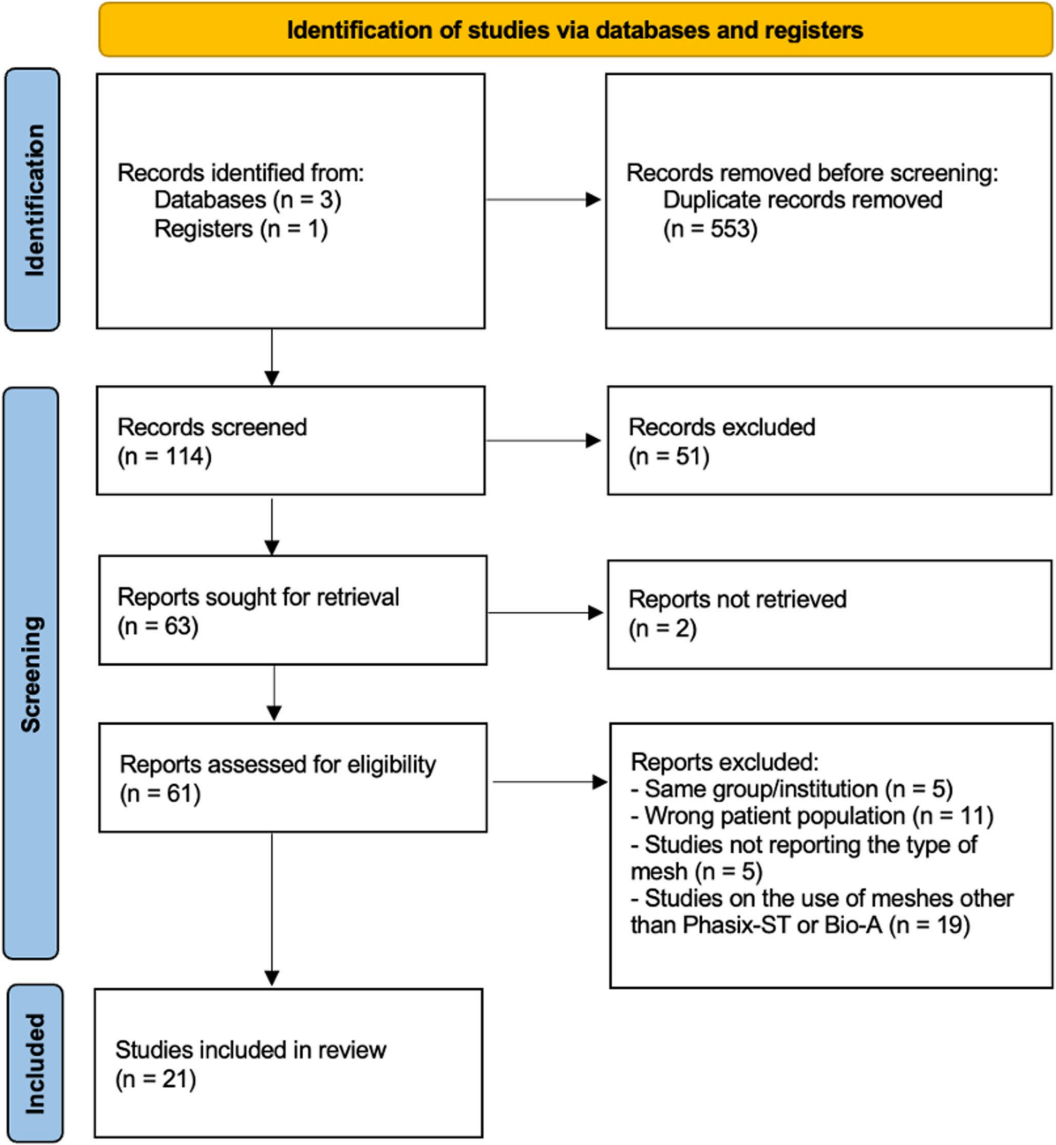
The Preferred Reporting Items for Systematic Reviews checklist (PRISMA) diagram

The PRISMA flowchart is presented in Fig. 1. The search yielded 667 results, which were reduced to 114 after duplicate removal. Following title and abstract screening, full-text articles were assessed for eligibility. At this stage, studies were excluded due to overlapping patient populations or the use of mesh types other than those specified [17–21]. Ultimately, 21 articles issued from 2012 to 2025 fully met the inclusion criteria and were included in the final analysis. Most studies were retrospective cohorts, with two prospective studies included [22, 23]. Six studies used Phasix™ ST mesh [22, 24–28], fourteen employed Gore Bio-A^®^ mesh [23, 29–41], and one retrospective study directly compared the two meshes [42]. The quality of the included studies is depicted in Supplementary Fig. 1. Two studies were identified as having a low risk of bias, ten were assessed as presenting a moderate risk of bias, and nine were determined to have a high risk of bias.

### Systematic review

Overall, 2208 patients were included for analysis. Phasix™ ST was used in 1035 (47%) while Gore Bio-A^®^ was adopted in 1173 subjects (53%). Demographic and clinical details are summarized in Table 1. The sample size of individual studies varied from 8 to 399 patients. Patients’ age ranged from 46 to 74 years, with 66% being female. Preoperative BMI ranged from 23 to 32 kg/m^2^. HH type was detailed in 14 articles, with 74% classified as type III-IV HH. Baseline characteristics were comparable between groups.

**Table 1:** Demographic and clinical characteristics of included patients. Study design retrospective (Ret); prospective (Pro); number of patients (No. pts); male (M); female (F); body mass index (BMI). Data are reported as numbers, mean ± standard deviation

Author	Year	Country	Study Design	Mesh type	No pts	Sex F/M	Age (years)	BMI (Kg/m^2^)	Hiatal hernia type I/II-III/IV	Primary / Recurrent
Ukegjini et al. [26]	2023	Switzerland	Ret	Phasix ST	97	59/38	65 ± 14	27 ± 4.5	4–80	84/13
Konstantinidis et al. [28]	2023	Greece	Ret	Phasix ST	30	12/18	56 ± 40	27.5 ± 13	nr	19/21
Siemssen et al. [27]	2024	Germany	Ret	Phasix ST	176	88/88	55 ± 10.5	nr	nr	176/0
Salehi et al. [25]	2024	USA	Ret	Phasix ST	308	191/117	61.4 ± 15.4	27.7 ± 4.8	nr	261/47
McKay et al. [22]	2024	USA	Pro	Phasix ST	50	32/18	65.5 ± 10	30.7 ± 5.4	nr	50/0
Fair et al. [24]	2024	USA	Ret	Phasix ST	230	171/59	63.6 ± 13.7	29.3 ± 4.8	nr	nr
Aiolfi et al. [42]	2025	Italy	Ret	Phasix ST	144	119/25	67.8 ± 11.3	27.3 ± 3.8	10–134	88/56
				Bio-A	127	103/24	67.1 ± 9	26.9 ± 3.3	15–112	99/28
Massullo et al. [32]	2012	USA	Ret	Bio-A	11	9/2	62.3 ± 36.5	31.7 ± 17.5	9 − 2	11/0
Powell et al. [33]	2013	USA	Ret	Bio-A	70	47/23	59.7	28.3	22–48	70/0
Gebhart et al. [36]	2013	USA	Ret	Bio-A	86	nr	57.3 ± 14.3	nr	0–86	75/9
Priego Jiménez et al. [34]	2014	Spain	Ret	Bio-A	10	7/3	66.8 ± 24.9	32.8 ± 10.7	0–10	8/2
Alicuben et al. [35]	2014	USA	Ret	Bio-A	114	65/49	66	nr	68 − 46	89/25
Silecchia et al. [23]	2014	Italy	Pro	Bio-A	10	9/1	52 ± 9.3	26.4 ± 2.4	9 − 1	10/0
Berselli et al. [40]	2015	Italy	Ret	Bio-A	8	7/1	73.7 ± 23.2	28.5 ± 12.3	0–8	8/0
Olson et al. [30]	2018	USA	Ret	Bio-A	399	261/138	59.6 ± 13.4	29.9 ± 5	nr	399/0
Iossa et al. [37]	2019	Italy	Ret	Bio-A	28	18/10	46 ± 23	23 ± 5	22 − 6	28/0
Borman et al. [29]	2019	USA	Ret	Bio-A	96	63/33	58.2 ± 14.8	30.3 ± 6.2	43–53	90/6
Arcerito et al. [41]	2020	USA	Ret	Bio-A	70	47/23	64 ± 51.9	nr	0–70	70/0
Tartaglia et al. [38]	2021	Italy	Ret	Bio-A	44	29/15	62 ± 52.6	24.5 ± 5.9	31 − 14	44/0
Armijo et al. [39]	2021	USA	Ret	Bio-A	83	37/46	57 ± 13.6	28.6 ± 3.8	nr	75/8
Ardu et al. [31]	2022	Italy	Ret	Bio-A	17	17/0	66.2 ± 9.7	28.5 ± 4.9	0–17	16/1

Surgical details are reported in Table 2. In total, 84% of procedures were conducted for primary HH, with 81% completed via laparoscopic approach. The technique for cruroplasty was documented across 16 studies, demonstrating heterogeneity that appeared to reflect individual surgeon preferences. Posterior cruroplasty was performed in 81.2% of cases, combined posterior and anterolateral hiatoplasty in 18.5%, and anterior repair was utilized in 0.3% of patients. Reporting of mesh shapes varied among studies; however, the U-shaped configuration was commonly observed in patients who underwent Bio-A^®^ buttressing, whereas Phasix™ ST mesh placement included Keyhole, reverse C, or U-shaped configurations. Fixation methods also differed across studies, encompassing non-absorbable sutures, absorbable sutures, pledgets, and glue. Details on fundoplication techniques were not consistently provided; nonetheless, the majority of studies indicated the use of Nissen (44%) or Toupet (34%) fundoplication. Notably, Salehi et al. and Ardu et al. reported cruroplasty alone without fundoplication [25, 31]. The mean operative time was comparable for Bio-A^®^ vs. Phasix™ ST mesh (range 70–223 min vs. 45–209 min), while conversion to open surgery occurred in 0.3% of patients. Pulmonary (3.3%), infectious (1.3%), cardiovascular (1.1%), and esophageal/gastric leak (0.6%) were the most commonly reported complications with no differences among Bio-A^®^ and Phasix™ ST.

**Table 2:** Operative characteristics of included patients. Cruroplasty posterior (P), anterior (A), posterior and anterior (PA); fixation method absorbable sutures (AS), non-absorbable sutures (NAS), absorbable tacks; antireflux procedure Nissen (N), Toupet (T), other (O), none (No); surgical approach laparoscopic (L), robotic (R), open (O); conversion to open surgery from laparoscopic/robotic approach (L/O). Data are reported as numbers, mean ± standard deviation

Author	No pts	Mesh type	Cruroplasty *P*-A-PA	Mesh shape	Fixation method	Antireflux procedure *N*-T-O-No	Operative time (min)	Surgical Approach L-*R*-O	Conversion L/*R*-O
Ukegjini et al. [26]	97	Phasix ST	14-3-80	Keyhole	AS	21-5-71-0	178 ± 221.3	89-4-4	2 − 0
Konstantinidis et al. [28]	30	Phasix ST	nr	Keyhole	AT or AS	30-0-0-0	72 ± 50.4	10-20-0	0
Siemssen et al. [27]	176	Phasix ST	66-0-110	Keyhole	AS	nr	45 ± 13	176-0-0	nr
Salehi et al. [25]	308	Phasix ST	308-0-0	Reverse C	NAS	0-0-0-308	126 ± 38.4	0-308-0	nr
McKay et al. [22]	50	Phasix ST	50-0-0	U shaped	AT ± pledgets	17-33-0-0	161	50-0-0	0
Fair et al. [24]	230	Phasix ST	nr	nr	NAS	nr	137.6 ± 38.7	209-21-0	nr
Aiolfi et al. [42]	144	Phasix ST	109-0-35	U shaped / Keyhole	NAS	0-144-0-0	139 ± 63	144-0-0	0
	127	Bio-A	101-0-26	U shaped	NAS	0-127-0-0	158 ± 49.6	127-0-0	0
Massullo et al. [32]	11	Bio-A	11-0-0	U shaped	AT	nr-nr-0-0	nr	11-0-0	nr
Powell et al. [33]	70	Bio-A	70-0-0	Squared	Glue	70-0-0-0	nr	70-0-0	nr
Gebhart et al. [36]	86	Bio-A	76-0-0	U shaped	NAS	76-0-0-10	88 ± 25	86-0-0	0
Priego Jiménez et al. [34]	10	Bio-A	10-0-0	U shaped	6 AT, 4 glue	10-0-0	140.7 ± 17.2	10-0-0	0
Alicuben et al. [35]	114	Bio-A	114-0-0	U shaped	AT, NAS, glue	76-38-0-0	nr	114-0-0	nr
Silecchia et al. [23]	10	Bio-A	10-0-0	Modified U shaped	AT ± glue	10-0-0-0	70 ± 1	10-0-0	0
Berselli et al. [40]	8	Bio-A	nr	U shaped	AS + glue	7-1-0-0	166.7 ± 107.2	8-0-0	0
Olson et al. [30]	399	Bio-A	nr	U shaped	AT	225-170-4-0	nr	399-0-0	4
Iossa et al. [37]	28	Bio-A	28-0-0	U shaped	AS + glue	28-0-0-0	90 ± 13	28-0-0	2
Borman et al. [29]	96	Bio-A	nr	nr	NAS	69-27-0-0	nr	96-0-0	nr
Arcerito et al. [41]	70	Bio-A	70-0-0	U shaped	NAS	59-11-0-0	223 ± 133	0-70-0	0
Tartaglia et al. [38]	44	Bio-A	44-0-0	U shaped	AS + glue	26-18-0-0	127 ± 37.8	44-0-0	0
Armijo et al. [39]	83	Bio-A	nr	U shaped	NAS	53-30-0-0	220 ± 220.3	83-0-0	0
Ardu et al. [31]	17	Bio-A	17-0-0	U shaped	NAS ± glue	8-2-2-5	nr	17-0-0	0

Postoperative data are detailed in Table 3. Mean follow-up duration was longer for Bio-A^®^ compared to Phasix™ ST (45.8 vs. 27.8 months). Although the definition of anatomical HH recurrence was heterogeneous across studies (i.e. more than 2 cm or any hernia size), the overall HH recurrence rate was 8.2% (*n* = 170 patients), with an estimated clinical trend toward higher incidence for Bio-A^®^ vs. Phasix™ ST mesh (10% vs. 6%). Overall, 34.8% of patients required surgical revision for HH recurrence with a trend toward higher rates for Bio-A^®^ vs. Phasix™ ST (36.3% vs. 19.3%). Overall postoperative complications occurred in 10.6% of patients (16 studies). With the exception of one isolated case of esophageal stenosis following HH repair with Bio-A^®^ mesh, which required percutaneous endoscopic gastrostomy and subsequent reoperation for recurrence two months postoperatively, no additional mesh-related complications were observed. Additionally, there were no mesh-related mortalities reported.

**Table 3:** Postoperative follow-up. Postoperative complications Clavien-Dindo ≥ 3 (CD ≥ 3); hospital length of hospital stays (HLOS). Data are reported as numbers, mean ± standard deviation

Author	No pts	Mesh type	Overall complications	Severe complications (CD *≥* 3)	HLOS (days)	Recurrence	Redo surgery	Follow-up (months)
Ukegjini et al. [26]	97	Phasix ST	15	3	4 ± 1.5	8	2	12 ± 12.8
Konstantinidis et al. [28]	30	Phasix ST	0	0	1	0	0	14 ± 15.6
Siemssen et al. [27]	176	Phasix ST	27	nr	2 ± 0.6	4	2	22 ± 5.2
Salehi et al. [25]	308	Phasix ST	nr	nr	nr	20	nr	26.8 ± 7.2
McKay et al. [22]	50	Phasix ST	0	0	2.8	8	0	63.6 ± 4
Fair et al. [24]	230	Phasix ST	nr	nr	nr	11	nr	20 ± 14.6
Aiolfi et al. [42]	144	Phasix ST	15	3	3 ± 0.7	11	2	51 ± 12.6
	127	Bio-A	16	2	3 ± 1.5	15	2	94 ± 15.6
Massullo et al. [32]	11	Bio-A	3	1	nr	1	0	13.4 ± 3.5
Powell et al. [33]	70	Bio-A	0	0	nr	0	0	3–18
Gebhart et al. [36]	86	Bio-A	10	8	2 ± 3	17	nr	30 ± 11
Priego Jiménez et al. [34]	10	Bio-A	3	0	3.3 ± 2.6	1	0	20.1 ± 17.2
Alicuben et al. [35]	114	Bio-A	6	3	nr	1	0	12
Silecchia et al. [23]	10	Bio-A	1	0	3 ± 1.2	0	0	17.4
Berselli et al. [40]	8	Bio-A	1	nr	11 ± 18.5	2	0	27.2 ± 26.8
Olson et al. [30]	399	Bio-A	nr	nr	nr	49	24	44.7 ± 22.8
Iossa et al. [37]	28	Bio-A	nr	nr	4 ± 3	2	0	41
Borman et al. [29]	96	Bio-A	nr	nr	nr	7	2	27.7 ± 7.6
Arcerito et al. [41]	70	Bio-A	6	1	nr	6	4	29 ± 32.6
Tartaglia et al. [38]	44	Bio-A	2	2	3 ± 8.9	2	0	36
Armijo et al. [39]	83	Bio-A	15	4	nr	13	nr	nr
Ardu et al. [31]	17	Bio-A	2	0	nr	2	0	42.5

### Meta-analysis

Anatomical hernia recurrence was reported in all included studies (2208 patients). The quantitative analysis showed no statistically significant difference for Bio-A^®^ vs. Phasix™ ST (RR = 1.29, 95% HPD 0.01–8.15). The between-study standard deviation across single-arm studies on each arm was τ = 0.44 (95% HPD 0.11–2.79) for Phasix™ ST and τ = 0.58 (95% HPD 0.10–2.25) for Bio-A^®^ trials, indicating reasonable heterogeneity. The linear correlation between arms was σ =-0.03 (95% HPD − 0.93–0.97).

Overall complications were reported in 16 studies (1147 patients). The quantitative analysis showed no statistically significant difference for Bio-A^®^ vs. Phasix™ ST (RR = 1.16, 95% HPD 0.01–8.75). The between-study standard deviation across single-arm studies on each arm was τ = 0.62 (95% HPD 0.09–3.79) for Phasix™ ST and τ = 0.64 (95% HPD 0.13–4.25) for Bio-A^®^ studies, indicating high heterogeneity. The linear correlation between arms was σ = -0.02 (95% HPD − 0.896–0.997). Similarly, severe postoperative complications (CD ≥ 3), were comparable for Bio-A^®^ vs. Phasix™ ST (RR = 1.06, 95% HPD 0.01–12.33). The between-study standard deviation across single-arm studies on each arm was 0.78 (95% HPD 0.11–4.79) for Phasix™ ST and 0.82 (95% HPD 0.05–5.67) for Bio-A^®^ trials, indicating high heterogeneity. The linear correlation between arms was σ = -0.72 (95% HPD − 0.994 − 0.992).

## Discussion

This study suggests that cruroplasty with Bio-A^®^ or Phasix™ ST mesh buttressing is safe with comparable overall, severe, and mesh-related complications. The overall recurrence rate for both Bio-A^®^ and Phasix™ ST was low. Despite the estimated clinical trend toward higher HH recurrences for Bio-A^®^, the quantitative analysis concluded no significant HH recurrence risk on medium-term follow-up.

HH is a progressive, multifactorial condition that compromises the physiological antireflux barrier. The prevalence of HH in the adult population is estimated at 15–20%, with type 3 and 4 hernias accounting for 5–10% of cases [2, 43]. Surgical management traditionally involves reduction of herniated stomach, repair of the crural defect, and restoration of the lower esophageal sphincter [44]. However, up to 15% of patients develop symptomatic recurrence requiring pharmacological therapy, outpatient visits, additional instrumental assessment, and in some cases revisional surgery with a significant effect on overall costs. Crural failure is identified as the primary driver for recurrence in 50–80% of cases, leading to stomach re-herniation and, eventually, fundoplication disruption [43, 45]. Consequently, recent research has prioritized the optimization of cruroplasty in both primary and revisional settings. Direct suture repair has consistently been associated with high recurrence rates in large HH, estimated between 20 and 40% depending on the definition of recurrence. This failure rate is biologically plausible given the complex tension forces acting on the suture line, which may weaken the crura integrity. Nguyen et al. reported significantly higher HR for recurrence in cases with tension on the suture line (HR 2.05, 95% CI 1.33–3.17, *p* = 0.001) [46]. Furthermore, the continuous dynamic movement of the diaphragm, combined with underlying connective tissue weakness, can compromise long-term suture integrity [4, 47, 48]. To address these issues, various optimization strategies have been proposed, including figure-of-eight sutures, “low tension triangulations” or tension relieving incisions, and the use of PTFE pledgets to provide broader grip on the crural tissue [4]. More recently, the use of autologous Platelet-Rich-Plasma to strengthen diaphragmatic crura has been proposed with promising preliminary results [49, 50]. Further, crural reinforcement with alternatively falciform ligament flap, posterior rectus sheath, or mesh has emerged as a strategy to buttress the repair in selected cases [51, 52]. Non-absorbable meshes have been described in the past with promising results in terms of recurrence minimization but with associated mesh-related complications and mortality [53]. Hence, biosynthetic absorbable meshes, acting as a scaffold for tissue ingrowth, have demonstrated safety and efficacy in minimizing related complications thus reducing short- and medium-term recurrence rates [8, 54–56]. Contemporary practice predominantly utilizes Phasix™ ST and Bio-A^®^ bioabsorbable meshes because of reduced perivisceral inflammatory response and limited fibrotic reaction. Recent studies and meta-analyses reported the safety profile of both Bio-A^®^ and Phasix™ ST. Specifically, Ganam et al. in their meta-analysis of 211 patients treated with Phasix™ ST observed low recurrence rates (OR = 5%; 95% CI 0.01–0.10) and low postoperative dysphagia rate (OR = 3%, 95% CI 0.00-0.10), with no reoperations over a median follow-up of 12–27 months [57]. Similarly, recent retrospective series by Salehi and Fair reported recurrence rates of 5–6% with no mesh-related complications [24, 25]. Regarding Bio-A^®^ mesh, Clapp et al. observed a statistically significant reduction in recurrence rate compared to non-mesh repair (8% vs. 18%; *p* < 0.001) at 27 months mean follow-up [58]. Similarly, a recent multicenter retrospective analysis compared the safety and effectiveness of Bio-A^®^ vs. Phasix™ ST with no significant differences in terms of HH recurrence [42]. However, given the limitations related to the relatively limited sample size and study design, the present analysis was performed in attempt to pool data from single-arm series thus providing more robust evidence. Specifically, analysing more than two thousand patients, our review identified an overall recurrence rate of 8.2% with a clinical trend toward a higher estimated incidence of anatomical recurrence for Bio-A^®^ compared to Phasix™ ST (10% vs. 6%). However, despite the point estimation was 1.29 thus suggesting a possible higher risk for HH recurrence for Bio-A^®^, the quantitative analysis concluded comparable results between the two groups (RR = 1.29, 95% HPD 0.01–8.15). Interestingly, the evaluation of heterogeneity across single-arm studies on each arm showed reasonable heterogeneity. Since data were reported as aggregated, a formal meta-regression analysis stratified for type of fundoplication, surgery for recurrence, mesh shape, and timing of follow-up was not feasible. Our findings seem in contrast with the network meta-analysis by Weiss et al., which reported a higher recurrence rate for non-absorbable mesh repair (34.7%) [59]. Conversely, Hanna et al. reported conflicting data, showing a lower recurrence risk (RR = 0.50, 95% CI 0.28–0.88) in observational studies meta-analysis, but no difference when considering randomized trials (RR = 0.98, 95% CI 0.47–2.02) [60]. Caution is advised when interpreting our findings due to potential selection and reporting bias, variability in the definition of HH recurrence, differences in follow-up duration (longer in Bio-A^®^), mesh shape, fixation techniques, cruroplasty methods, and choice of fundoplication [61, 62]. The absence of a standardized definition for HH recurrence represents a notable gap in the current literature. Although SAGES guidelines recommend defining recurrence as an objective defect greater than 2 cm accompanied by symptoms, many studies utilize unvalidated symptom scores or lack independent objective assessments such as endoscopy, pH monitoring, or manometry [3, 63, 64]. Recent European consensus indicates that recurrence should be identified when more than 2 cm of gastric tissue is found above the diaphragm alongside recurring GERD-related symptoms [65]. Finally, it is important to distinguish the anatomical location of the diaphragmatic defect, as posterior migration might suggest a true recurrence, whereas anterior enlargement of the esophageal hiatus might suggest a progression of the disease rather than a real recurrence [48, 66].

Postoperative complications were documented in 16 studies, revealing an overall incidence of 10.6%. Quantitative analyses demonstrated that both mesh types exhibit similar risk profiles, with no statistically significant differences observed between groups for overall (RR = 1.16) or severe postoperative complications (RR = 1.06). Notably, heterogeneity was high, which may indicate variability in outcome reporting, potential under reporting or detection bias. Mesh-related complications possibly attributable to technical issues were rare, occurring in only one patient who developed esophageal stenosis necessitating percutaneous gastrostomy and subsequent revisional surgery at two months postoperatively. Biosynthetic absorbable mesh appears to be well tolerated, providing structural durability and tissue biocompatibility while promoting a reduced inflammatory response and minimized perivisceral fibrosis. These findings are consistent with those of Hanna et al., who reported complication rates comparable to cruroplasty alone (RR = 1.36, 95% CI 0.69–2.69). Likewise, Campos et al. identified a 1.5% rate for postoperative complications, and Muller-Stich reported a favourable safety profile for mesh reinforcement, with mesh-associated complications at 1.9%, comparable to non-mesh repair (OR = 1.02; *p* = 0.94) [60, 67].

The findings of this analysis are subject to some limitations inherent to the nature of the included literature. Most studies were retrospective single-arm cohorts, introducing potential selection and publication biases; although a Bayesian approach with hierarchical power priors was employed to mitigate the lack of head-to-head studies, the strength of causal inferences remains constrained. Significant clinical heterogeneity exists regarding surgical techniques, hospital volume and surgeon expertise, variations in mesh configuration (U-shaped vs. keyhole vs. reverse C), fixation methods (sutures, tacks, or fibrin glue), and fundoplication type (Nissen vs. Toupet) [68, 69]. Since data were heterogeneously detailed or reported as aggregated, a formal meta-regression analysis with stratification of results resulted unfeasible. Furthermore, the absence of a standardized definition for recurrence limits the comparability of outcomes across series. Finally, the variability in follow-up duration, often restricted to the short-to-medium term, precludes definitive conclusions regarding the long-term anatomical durability and possible attrition bias. However, this reflects the recent adoption of biosynthetic meshes.

## Conclusion

This meta-analysis suggests that crural reinforcement using either Bio-A^®^ or Phasix™ ST synthetic absorbable mesh appear safe, with no significant differences noted in overall, severe, or mesh-related complications. Both meshes seem to demonstrate potential for reducing HH recurrence in medium-term follow-up. However, long-term durability remains uncertain, highlighting the need for future studies focusing on extended follow-up.

## Supplementary Information

Supplementary Material 1: Supplementary Figure 1. Quality assessment of the included studies (ROBINS-I tool). Each domain is evaluated with one of the following: low (green circle), moderate (yellow circle), serious (red circle), critical, NI (no information) (TIFF 5.84 MB).

## Data Availability

The data collected and analysed during the current review are available from the corresponding author on reasonable request.
