# Supplementary figures and images for: Efficacy and safety of mesh reinforced cruroplasty with Phasix™ ST vs. Bio-A®: systematic review and bayesian meta-analysis
Source: Hernia. 2026 Apr 15;30(1):158. doi: 10.1007/s10029-026-03666-y (PMC13083453; doi:10.1007/s10029-026-03666-y)

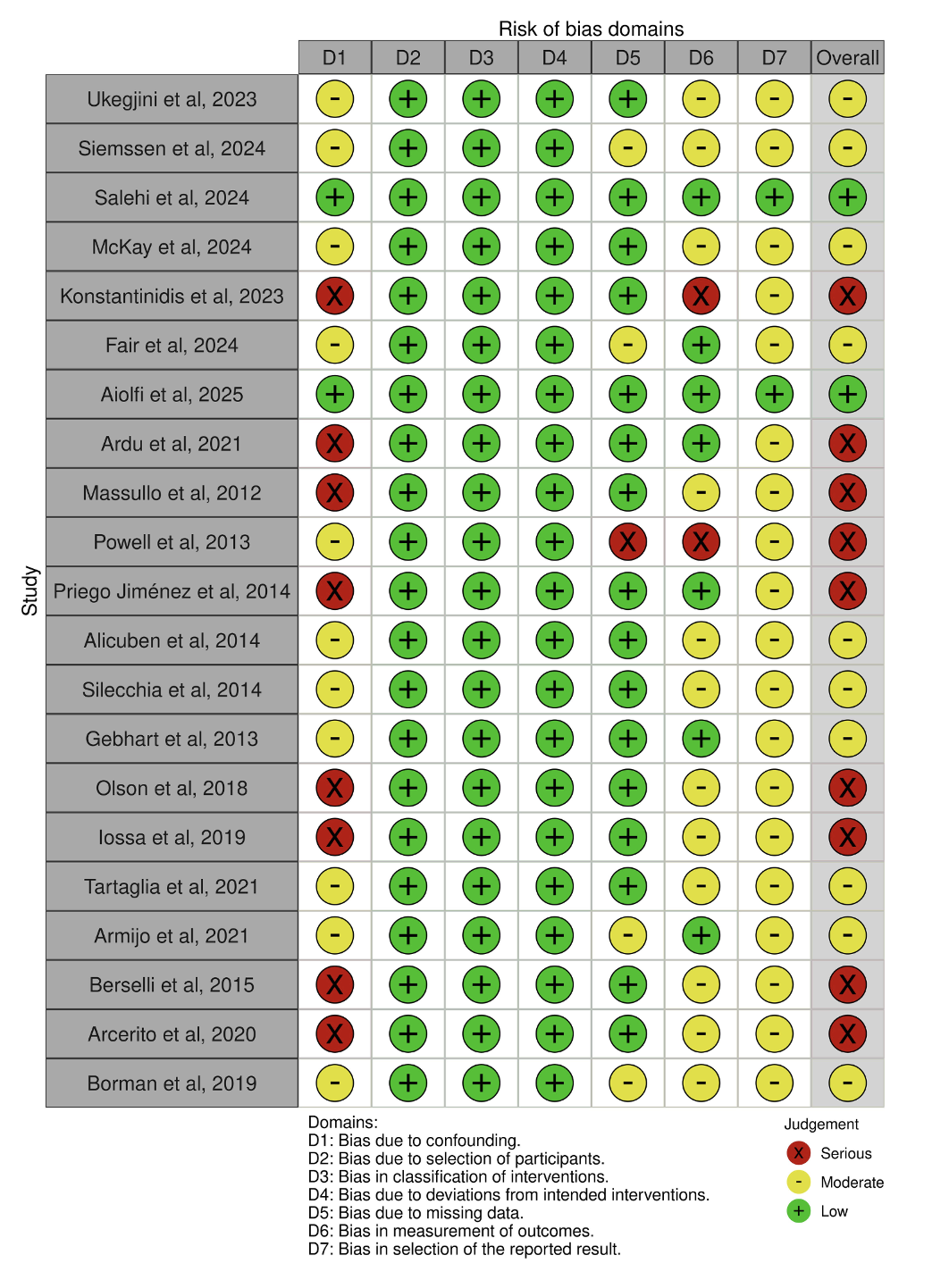

Supplement: Supplementary file 1 — Supplementary Material 1: Supplementary Figure 1. Quality assessment of the included studies (ROBINS-I tool). Each domain is evaluated with one of the following: low (green circle), moderate (yellow circle), serious (red circle), critical, NI (no information) (TIFF 5.84 MB). [file 10029_2026_3666_MOESM1_ESM.tiff]
